# Is Misdiagnosis of Type 1 Diabetes Mellitus in Malaysian Children a Common Phenomenon?

**DOI:** 10.3389/fendo.2021.606018

**Published:** 2021-03-08

**Authors:** Meenal Mavinkurve, Muhammad Yazid Jalaludin, Elaine Wan Ling Chan, Mazidah Noordin, Nurshadia Samingan, Annie Leong, Azriyanti Anuar Zaini

**Affiliations:** ^1^Department of Paediatrics, School of Medicine, International Medical University, Wilayah Persekutuan, Kuala Lumpur, Malaysia; ^2^Department of Paediatrics, University Malaya Medical Centre, Kuala Lumpur, Malaysia; ^3^Department of Paediatrics, Faculty of Medicine, University Malaya, Kuala Lumpur, Malaysia; ^4^Institute for Research, Development and Innovation, International Medical University, Kuala Lumpur, Malaysia; ^5^Department of Paediatrics, School of Medicine, University Teknologi MARA, Selangor, Malaysia

**Keywords:** misdiagnosis, children, type 1 diabetes, diabetic ketoacidosis, Malaysia

## Abstract

**Background:**

Children with Type 1 diabetes (T1DM) commonly present in diabetic ketoacidosis (DKA) at initial diagnosis. This is likely due to several factors, one of which includes the propensity for T1DM to be misdiagnosed. The prevalence of misdiagnosis has been reported in non-Asian children with T1DM but not in Asian cohorts.

**Aim:**

To report the rate of misdiagnosis and its associated risk factors in Malaysian children and adolescents with T1DM.

**Methods:**

A retrospective analysis of children with T1DM below 18 years of age over a 10 year period was conducted.

**Results:**

The cohort included 119 children (53.8% female) with a mean age 8.1 SD *± 3.9* years. 38.7% of cases were misdiagnosed, of which respiratory illnesses were the most common (37.0%) misdiagnosis. The rate of misdiagnosis remained the same over the 10 year period. Among the variables examined, younger age at presentation, DKA at presentation, healthcare professional (HCP) contact and admission to the intensive care unit were significantly different between the misdiagnosed and correctly diagnosed groups (p <0.05).

**Conclusion:**

Misdiagnosis of T1DM occurs more frequently in Malaysian children <5 years of age. Misdiagnosed cases are at a higher risk of presenting in DKA with increased risk of ICU admission and more likely to have had prior HCP contact. Awareness of T1DM amongst healthcare professionals is crucial for early identification, prevention of DKA and reducing rates of misdiagnosis

## Introduction

Type 1 diabetes (T1DM) is a common autoimmune condition of childhood with a peak age of onset at 10–14 years of age (1). The incidence of T1DM varies worldwide, being higher in Northern Europe. T1DM is the commonest form of childhood diabetes in Malaysia, accounting for 73%–77% of all childhood diabetes cases as reported by the DiCARe registry (1–3). The International Diabetes Federation reported 977 cases of T1DM in Malaysian children aged 0–19 years in 2019 (4).

Timely diagnosis of T1DM is essential to initiate prompt treatment and avoid the progression to diabetic ketoacidosis (DKA), which occurs in 30%–70% of children (3, 5). The incidence of DKA at initial diagnosis is influenced by younger age (<5 years old) at presentation, lower socioeconomic status, lower parental education, lack of parental and health practitioner awareness of the symptoms of diabetes, limited access to healthcare and a lower prevalence of childhood T1DM (6). In Malaysia, 64.2% of children with T1DM present in DKA at initial diagnosis (3).

There is a positive correlation between misdiagnosis of T1DM and DKA (7, 8). This may be due to the fact that the presenting features of T1DM overlap with symptoms of more common childhood diseases such as respiratory, gastrointestinal, and surgical illnesses (1, 9). Furthermore, if the osmotic symptoms are not forth-coming in the medical history, T1DM may not be considered as a potential diagnosis. Some studies have reported on the risk factors which contribute to misdiagnosis of diabetes in North-American and European children, however, there are no published studies on the rates of misdiagnosis and its associated factors in Asian countries (10, 11). The aim of this study is to report on the rate of misdiagnosis of T1DM in Malaysian children and adolescents and the risk factors which predispose to misdiagnosis.

## Methods

A retrospective review was conducted on all newly diagnosed cases of paediatric T1DM who presented to University Malaya Medical Centre (UMMC) between January 1^st^ 2010 and December 31^st^ 2019. A diagnosis of T1DM was made according to the ISPAD guidelines for the year in which the diagnosis was made, as was diabetic ketoacidosis (DKA) (12–14). Misdiagnosis was defined as any subject who had been given a diagnosis *other than diabetes* or DKA, either by the referring physician or by the doctor at UMMC. Children with diabetes mellitus other than T1DM or those cases in which misdiagnosis could not be confirmed were excluded from the analysis. Data was extracted from the electronic medical record (EMR) system and the referral letters from the referring physicians. Details on age, sex, ethnicity, symptoms, and contact with a health care professional prior to the initial diagnosis were obtained. Additional details on anthropometry and biochemistry were also extracted. UMMC has an EMR system with a proforma for in-patient admission clerking, into which details on the presenting history are entered on admission. All new diagnoses of paediatric T1DM at UMMC are admitted as inpatients irrespective of whether they present in DKA or not and all are reviewed by the Paediatric Endocrinology team.

Statistical analysis was done using SAS@ 9.4 software (SAS Institute, Cary NC). The demographic data, clinical and laboratory features, were analyzed using descriptive statistics. Data are expressed as the median with interquartile range (IQR) or means for continuous variables and frequencies/percentages for categorical variables. Comparisons were made between the misdiagnosed and correctly diagnosed groups for demographic and clinical variables using T-test (two continuous group comparison); and chi-square/Fisher-exact test (for categorical group comparison). A p-value of less than 0.05 was accepted as being statistically significant.

This study was approved by the University Malaya Medical Centre (UMMC) institutional ethics board MREC Ref: 2019325-7251.

## Results

A total number of 223 cases of childhood diabetes were newly diagnosed between January 1^st^ 2010 and December 31^st^ 2019 at UMMC. Out of these, 62% (n=138) were T1DM, 35% (n=77) T2DM and 4% (n=8) other diabetes. Of the 138 T1DM cases during the study period, 19 were excluded, as misdiagnosis could not be confirmed based on information entered into the EMR system. Seventy-three cases (53.8%) were correctly diagnosed as T1DM, whereas 46 (38.7%) were *misdiagnosed*.

### Demographics

The mean age at diagnosis was 8.1 years (± 3.9). The majority, 47.1% (n=56), were aged 5-10 years and 53.8% (n=64) were female. Malay ethnicity representation was 35.6% (n=42), Chinese 32.8% (n=39) and Indian 21.0% (n=25). The majority 63.0% (n=70) of the referrals came from within the UMMC coverage area of 450 sq. kilometres with a 25 km radius within the Klang valley region in the states of Selangor and W.P. Kuala Lumpur. **Table 1** illustrates the demographic and clinical characteristics of the T1DM patients.

**Table 1:** Demographic and clinical characteristics of the overall cohort.

Characteristics	Participants (n = 119) (%)
**Age (years)**	
Mean (± SD)	*8.1 (± 3.9)*
**Age group (years)** (n= 119)	
<5	*25 (21.0)*
5–10	*56 (47.1)*
>10	*38 (31.9)*
**Gender** (n=119)	
Male	*55 (46.2)*
Female	*64 (53.8)*
**Ethnicity** (n=119)	
Malay	*42 (35.6)*
Chinese	*39 (32.8)*
Indians	*25 (21.0)*
Others	*13 (10.9)*
**Location of residence** (n=111)	
Within Hospital coverage	*70 (63.1)*
Outside Hospital Coverage	*41 (36.9)*
**BMI SDS** (n=90)	
Underweight	17 (18.9)
Normal	61 (67.8)
Overweight	12 (13.3)
**Blood glucose level (mmol/L)** (n=104)	
Mean (± SD)	27.0 (± 8.9)
**HbA1c at diagnosis (%)** (n=110)	
Mean (± SD)	12.3 (± 2.5)
**pH** (n=83)	
Mean (± SD)	7.0 (± 0.2)
**Presence of DKA** (n=115)	
Yes	82 (71.3)
No	33 (28.7)
**Severity of DKA** (n=75)	
Mild	15 (20.0)
Moderate	18 (24.0)
Severe	42 (56.0)
**Healthcare professional contact (HPC)** (n=118)	
1 HPC	90 (76.3)
2 HPC	21 (17.8)
>2 HPC	7 (5.9)
**Referral source** (n=112)	
Hospital Settings Primary Care Settings	77 (68.8) 35 (31.2)
**Admission to PICU** (n=94)	
Admitted	47 (20.0)
**Length of hospital stay** (n=99)	
Mean (± SD)	7.2 (± 2.7)

In terms of anthropometric measures, 67.8% (n=61) had normal weight, 19% (n=17) were underweight and 13.3% (n=12) were overweight at presentation. The mean HbA1c at presentation was 12.3% (± 2.5%) and 71.3% (n=82) had presented in DKA. Of those who had presented in DKA, 56% (n=42) were severe DKA. In terms of HCP contact prior to the initial diagnosis of T1DM, 76.3% (n=90) were seen by one healthcare professional prior to referral to UMMC. Referrals from other hospitals accounted for 68.8% (n=77) of the cases to UMMC. Paediatric ICU admission was needed in 20% (n=47) of the T1DM cases.

### Misdiagnosed Cases

**Figure 1:**
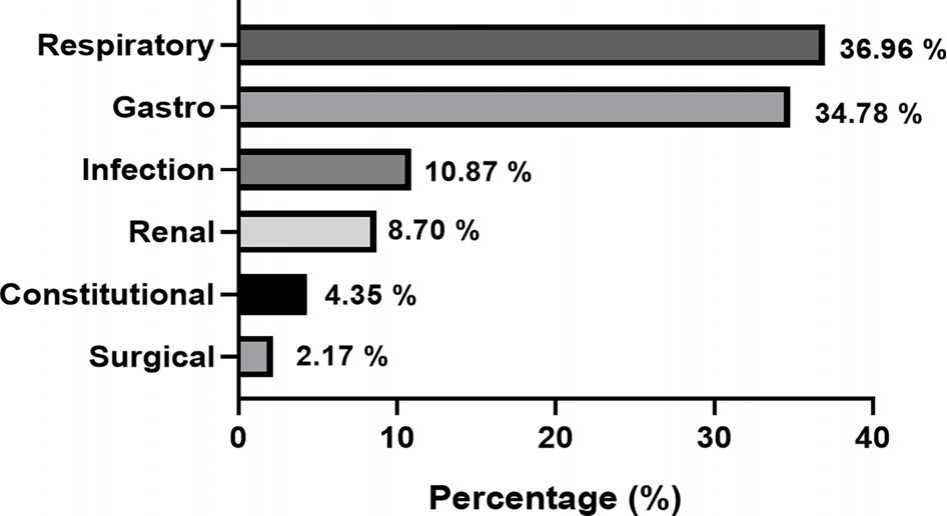
Categories of conditions made in T1DM children who were misdiagnosed.

The rate of *misdiagnosis* in this cohort was 38.7% (n=46). The most commonly reported symptoms were polyuria (45.7%), polydipsia (43.5%), weight loss (32.6%) and vomiting (32.6%). Symptoms such as skin infection, headache, polyphagia were reported in less than 5% of cases. The most common erroneous diagnoses made in children presenting with T1DM, were respiratory (36.9%), gastrointestinal (34.8%) and infectious (10.9%) illnesses. The rate of DKA in the misdiagnosed group was 87% (n=40). **Figure 1** depicts the categories of misdiagnoses made according to system in the T1DM cohort.

Regarding referral source, the total number of visits to primary care made just prior to final diagnosis of T1DM was (n=40). Of these, 52.5% (n=21) were misdiagnosed. The total number of visits made to either secondary or tertiary care institutions was (n=83), and of these 32.5% (n=27) were misdiagnosed.

The rate of misdiagnosis over the 10 year period varied between 20-64% with the highest rate being reported in 2019. Between 2012–2017, the rate of misdiagnosis of T1DM remained the same between 26%–40%. In 2018–2019, of those that were misdiagnosed (n=14), 65% (n=9) were diagnosed by primary care and 36% (n=5) by hospitalists. The increase in misdiagnosed cases between these two time periods was not contributed to by an overrepresentation of younger children (<5 years), as 29% were misdiagnosed during 2018–2019 and 33% during 2010–2017. During 2018–2019, the majority of cases were in the age group >5 years; 71% (n=10).

### Comparison of the Misdiagnosed and Correctly Diagnosed Cases of Childhood T1DM

No significant differences were found in terms of the mean age, gender or ethnicity between the 2 groups, as shown in **Table 2**. However, there was a higher representation of children aged <5 years old in the misdiagnosed group (56% vs. 44%, p=0.028). No significant differences in BMI-SDS, mean blood glucose, mean HbA1c, or mean pH at diagnosis were found between the two groups. Of the children that presented in DKA, 48.8% (n=40) of cases were from the misdiagnosed group. Conversely, 84.9% (n=28) of children from the correctly diagnosed group had a *non*-DKA presentation. The odds ratio for DKA at initial diagnosis in the misdiagnosed group was 5.3 (CI 1.5748–15.1724, p 0.0008). No significant difference was found in terms of severity of DKA between the two groups. In the misdiagnosed group, 89.3% of cases were seen by ≥ 2 HCP prior to the initial diagnosis of T1DM (p 0.0001) at our centre. A significantly higher percentage of the misdiagnosed cases were referred from primary care doctors (57.1% vs. 42.9%, p 0.0136). Admission to the paediatric intensive care unit was more frequent in the misdiagnosed group (57.5% vs. 42.6, p=0.001) but no difference in terms of mean length of hospital stay was found. Comparisons of variables between the misdiagnosed and correctly diagnosed children are shown in **Table 2**.

**Table 2:** Comparison of the demographic and clinical characteristics of the misdiagnosed and correctly diagnosed T1DM groups.

Characteristics	Misdiagnosed N (%)	Correctly Diagnosed N (%)	p value
**Age (years)**			
Mean (± SD)	7.7 (± 4.1)	8.4 (± 3.8)	0.5998
**Age group (years)**			
<5	14 (56.0)	11 (44.0)	0.0288*
5 - 10	15 (26.8)	41 (73.2)	
>10	17 (44.7)	21 (55.3)	
**Gender**			
Male	22 (40.0)	33 (60.0)	0.7801
Female	24 (37.5)	40 (62.5)	
**Ethnicity**			
Malay	21 (50.0)	21 (50.0)	0.3139
Chinese	13 (33.3)	26 (66.7)	
Indians	8 (32.0)	17 (68.0)	
**BMI SDS**			
Underweight	9 (52.9)	8 (47.1)	0.9222
Normal	29 (47.5)	32 (52.5)	
Overweight	6 (50.0)	6 (50.0)	
**Blood glucose (mmol/L)**			
Mean (± SD)	28.0 (± 9.2)	26.4 (± 8.8)	0.7114
**HbA1c at diagnosis (%)**			
Mean (± SD)	11.9 (± 2.3)	12.6 (± 2.6)	0.4694
**pH**			
Mean (± SD)	7.0 (± 0.2)	7.1 (± 0.2)	0.8448
**Presence of DKA**			
Yes	40 (48.8)	42 (51.2)	0.0008*
No	5 (15.2)	28 (84.9)	
**Severity of DKA**			
Mild	4 (26.7)	11 (73.3)	0.0535
Moderate	8 (44.4)	10 (55.6)	
Severe	26 (61.9)	16 (38.1)	
**Healthcare professional contact (HPC)**			
0-1 HPC	21 (23.3)	69 (76.7)	0.0001*
≥2 HPC	25 (89.3)	3 (10.7)	
**Referral source**			
Primary Care Settings	20 (57.1)	15 (42.9)	0.0136*
Hospital Settings	25 (32.5)	52 (67.5)	
**Admission to PICU**			
Admitted	27 (57.5)	20 (42.6)	0.0017*
**Length of hospital stay**			
Mean (± SD)	7.8 (± 2.5)	6.8 (± 2.8)	0.4415

*P for all tests <0.05; T- test was used for two group comparison; and chi-square/Fisher- exact tests were used for categorical group comparison.

## Discussion

Our study shows that Malaysian children with T1DM have a misdiagnosis rate of 38.7%. Children are commonly diagnosed with alternative diagnoses, such as respiratory or gastrointestinal illnesses and despite the presence of osmotic symptoms, one-third of children are misdiagnosed. Younger children, < 5 years old, are more frequently misdiagnosed. As a result of the misdiagnosis, there is a higher risk of presenting in DKA and requiring ICU admission. Misdiagnosed cases are frequently referred from primary care services and more likely to have had ≥2 prior HCP contacts. To our knowledge, this is the first study to report on the prevalence of misdiagnosis in T1DM in Malaysian children.

The high rates of misdiagnoses (38.7%) were sustained with no reduction in rates during the study period. This is higher than the misdiagnosis rate of 16% reported by Munoz et al. (10) in American children <18 years. Methodological differences such as larger sample size, use of questionnaires may explain the discrepancy in rates of misdiagnosis. The use of questionnaires rather than accessing healthcare records for data collection means that ascertainment of a *“true”* misdiagnosis is difficult to make. Finally, the survey was conducted on participants from North America, where T1DM in children is more prevalent and hence greater public awareness of the disease as compared to Malaysia (4, 15, 16).

Respiratory, gastrointestinal conditions, infective and renal conditions were the commonest misdiagnoses given to T1DM children in this study. Other studies have similarly reported that infective conditions are common misdiagnoses in children with T1DM and this may be attributed to the fact that HCPs have greater exposure to common childhood illnesses rather than T1DM (10). A lack of familiarity amongst HCPs with the symptoms of childhood T1DM can lead to an erroneous diagnoses such as asthma, pneumonia and gastroenteritis, in which presenting symptoms are common with T1DM (1). This lack of familiarity is also evident from the finding that over one-third of misdiagnosed patients reported the presence of polyuria and polydipsia, which are classical symptoms of diabetes. The results also demonstrate that there is a lack of awareness about less common symptoms of diabetes in children, such as weight loss which was reported in one third of the misdiagnosed cases. The findings highlight the need for heightened awareness amongst HCPs about childhood T1DM and its presenting features and future HCP training should focus on improving these competencies (12, 17).

This study showed that there was a significantly higher rate of misdiagnosis in children < 5 years of age, as compared to those children aged > 10 years. These findings are similar to the study by Munoz et al. (10) which reported a misdiagnosis rate of 21% in the 0–6 year age group; 15% in 7–12 year and 14% in the 13-17 year age groups. Usher-Smith et al. (7) also reported a higher risk of diagnostic error in younger children with T1DM and a mean age 5.4 years. HCPs in our system need to have an increased awareness of the symptoms of diabetes in younger children and a higher index of suspicion for children who present with symptoms that are overlap between common childhood illnesses and T1DM. This may be achieved through continuing medical education on childhood diabetes topics or increasing clinical exposure to childhood diabetes cases.

The rate of DKA in the misdiagnosed group was higher (48.8%) as compared to the correctly diagnosed group. DKA was also 5.3 times more likely to occur in cases that were misdiagnosed. Though no significant differences in the severity of DKA were found we did show a significantly higher frequency of PICU admission from the misdiagnosed group. Munoz et al. (10) has also demonstrated that DKA was diagnosed in 68% of children with T1DM who were misdiagnosed as compared to only 42.8% in those who were correctly diagnosed. Similarly, a large systematic review by Usher-Smith et al. (7) reported that misdiagnosis is associated with a 3-fold increased risk of developing DKA. The relationship between misdiagnosis of childhood T1DM and the increased risk of DKA may be explained by various factors such as parental and HCP unfamiliarity with diabetes symptoms (7, 8, 11). Diminished parental awareness of the symptoms of childhood T1DM as demonstrated in a survey conducted by Diabetes UK highlighted that only 9% of British parents can correctly identify symptoms of childhood diabetes (18). Lack of awareness amongst parents or HCPs will undoubtedly put the child at risk of attending several HCPs thus contributing to a delay in diagnosis, and potentially leading to DKA (1, 9, 11). In our study, the referral patterns indicate that there is general lack of awareness of childhood T1DM amongst HCPs in Malaysia, as there was an over-representation of misdiagnosed cases being referred from the primary care setting as compared to hospitalist referrals. Furthermore, a large proportion of misdiagnosed children were seen by ≥2 HCPs prior to referral. A study by Pawlowicz et al. reported a similar finding in Poland, whereby 79.1% of erroneous diagnoses in childhood T1DM were made by primary care physicians as compared to 16.7% made by hospitalist doctors (5). This lack of awareness may be explained by factors such as limited exposure to childhood T1DM cases, which may either be during the post-graduate training or subsequent clinical exposure during practice. At present, shared care of paediatric diabetes patients between paediatricians and family medicine physicians does not exist in Malaysia. Furthermore, low regional prevalence rates of childhood T1DM may also be contributory factor for misdiagnosis. Hong et al. (3) has shown that DKA rates at initial presentation of paediatric T1DM in Malaysia is as high as 64%, much higher than in Northern European countries where prevalence rates of T1DM are higher (19–21).

Our data suggests that curbing DKA rates and admission to costly intensive care services in childhood T1DM hinges on making an accurate and early diagnosis. This can be achieved by raising public awareness and improving the competencies of HCPs in relation to childhood T1DM. With increased awareness, parents are more likely to seek medical attention early and HCPs to seek early referral. Public awareness campaigns, by means of education sessions, information posters, newspaper adverts and radio adverts have proven effective at reducing rates of DKA in Italy, the UK and Australia (22–25). The Parma study, showed that a large scale public awareness campaign can reduce the incidence of paediatric DKA from 78% to 12.5% (22). Similarly, the *4T’s* campaign in the UK has also contributed to reducing paediatric DKA rates (24, 25). It would stand to reason that similar interventions would reduce the rate of *misdiagnosis* of T1DM in Malaysia.

Though, in the opinion of the authors, this is the first study to report on the prevalence of misdiagnosis in T1DM in Malaysian children, we acknowledge its limitations. Firstly, the study is retrospective in nature. Secondly, the study is from a single centre which mainly covers an urban catchment area, and thus not necessarily reflective of national rates. Thirdly, we also acknowledge the small sample size. Future studies should capture data on larger numbers at a national level and include analyses on family and social factors that can influence rates of misdiagnosis.

In conclusion, misdiagnosis of T1DM in Malaysian children is a common phenomenon. Children under the age of 5 years are particularly at risk of being misdiagnosed. Misdiagnosis of T1DM predisposes to an increased risk of presenting in DKA and the need for increased healthcare resources. A significant number of these referrals are from the primary care setting highlighting the need for improving awareness of childhood T1DM amongst HCPs and the public in a region where T1DM is not a common childhood condition.

## Data Availability Statement

The raw data supporting the conclusions of this article will be made available by the authors, without undue reservation.

## Ethics Statement

This study was approved by the University Malaya Medical Centre (UMMC) institutional ethics board MREC Ref: 2019325-7251. Written informed consent for participation was not required for this study in accordance with the national legislation and the institutional requirements.

## Author Contributions

NS, MN, AL, and MM contributed to data collection. MM and AA initiated and designed the audit project. EC and MM did statistical analysis. MM analyzed and wrote the paper. AA and MJ corrected and edited the manuscript. All authors contributed to the article and approved the submitted version.

## Funding

Funding assistance for article processing charges came from Institute for Research, Development and Innovation (IRDI), International Medical University.

## Conflict of Interest

The authors declare that the research was conducted in the absence of any commercial or financial relationships that could be construed as a potential conflict of interest.
